# No clinical consequence of liner malseating in dual-mobility THAs at short term: a systematic review

**DOI:** 10.1007/s00402-023-04855-5

**Published:** 2023-04-10

**Authors:** Daniel Karczewski, Octavian Andronic, Doruk Akgün, Siegfried Adelhoefer, Philipp Kriechling, Henrik Bäcker

**Affiliations:** 1 Department of Orthopaedic Surgery, Musculoskeletal Oncology Service, Massachusetts General Hospital-Harvard Medical School, 55 Fruit Street, Boston, MA 02114 USA; 2 Department of Orthopaedic Surgery and Traumatology, Charité Berlin, University Hospital, Chariteplatz 1, 10117 Berlin, Germany; 3 Department of Orthopaedics, Balgrist University Hospital, University of Zürich, Forchstrasse 340, 8008 Zurich, Switzerland

**Keywords:** Dislocation, Hip instability, Metal ion, Trident liner, G7 liner

## Abstract

**Background:**

Liner malseating is well described in ceramic-on-ceramic total hip arthroplasties (THAs). However, limited information is known on this complication among dual-mobility articulations. As such, this systematic review analyzed liner malseating in dual-mobility THAs concerning prevalence, clinical implications, and associated risk factors.

**Methods:**

A PRISMA criteria-based systematic review was performed, and PubMed, Web of Science, MEDLINE, and Cochrane used as data bases. All original studies from 1980 to 2022 were considered eligible for inclusion, and Methodological Index for Nonrandomized Studies (MINORS) used for quality assessment.

**Results:**

In total, five retrospective cohort studies with 2330 patients (2673 dual-mobility THAs) were included. Mean age was 66.9 years, mean BMI was 29.8 kg/m2, and 35% of patients were female. Rates of malseating ranged from 0.15% to 5.8%, with a total of 53 malseated liners identified throughout all studies (1.98%). Based on THA manufacturer, malseating occurred in 48 Stryker (1.96%) and 5 Biomet Zimmer (2.14%) THAs. Mean clinical follow-up was 2.2 years (mean range, 1.3 to 6.4 years). Except one patient reporting of pain at 2 years, no revision or negative clinical implication was noted in any of the malseated liners, including normal ranged metal ions measured in four cases. A smaller acetabular component size was identified as a statistically significant risk factor for malseating in one study. Mean MINORS score was 9.8.

**Conclusions:**

Liner malseating is a rare finding in patients undergoing THAs with dual-mobility articulations. While prelim results demonstrate no negative clinical consequences to date, existing studies are limited, refer to short-term outcomes only, and do not prospectively follow-up affected patients.

Level of evidence: IV.

## Background

Total Hip Arthroplasty (THA) is widely considered the surgery of the century [1] with number of primary THAs projected to increase by 71% annually through 2030, in the United States alone [2]. However, dislocation remains one of the most common complications with up to 2% of THAs experiencing prosthetic hip dislocation within the first postoperative year [3]. The dual-mobility articulation, developed in 1976 by French physician Giles Bosquet, has become increasingly popular in recent decades [4], allowing for reduced rates of dislocation compared to conventional designs, while demonstrating implant survivorship rates of over 90% at midterm [5].

Nonetheless, dual-mobility designs are not without complications, including intraprosthetic dislocation, metallosis, liner fracture, and liner malseating [6]. The latter has been described in detail in the context of ceramic-on-ceramic THAs with malseating rates ranging over 20% [7], while resulting in significant clinical complications [8]. However, limited is known on liner malseating among dual-mobility THAs, as well as potential clinical implications [6]. As such, we analyzed liner malseating in dual-mobility THAs concerning overall malseating rate, patient characteristics, clinical and functional implications, as well as associated risk factors in the first systematic review to date.

## Patients and methods

The systematic review was performed on the basis of the PRISMA criteria (Preferred Reporting Items for Systematic Reviews and Meta-analyses) [9], and PubMed, Web of Science, Ovid Medline, and Cochrane used as data bases. Search criteria were set as: “(liner malseating OR dual-mobility liner malseating OR liner malseating hip arthroplasty)”. Inclusion criteria were: (1) Patients treated with a dual-mobility THA, (2) between 1980 and 2022, (3) and evaluation for liner malseating. Exclusion criteria were: (1) non-dual-mobility THAs, (2) experimental studies, and (3) non-English full texts. The search was performed by two independent reviewers (DK, HB). Duplicated search results were removed, and the remaining articles analyzed based on title, and if considered eligible as full text.

The quality assessment was performed using the Methodological Index for Nonrandomized Studies (MINORS) score independently applied by the same two reviewers, and a final score between 0 and 16 reached by consensus [10]. In addition, localization, publication year, study type, and level of evidence based on Ackley et al. [11] were analyzed for the purpose of quality and potential bias assessment. Outcome parameters included number and characteristics of dual-mobility THAs, number of patients and their baseline demographics (sex, age, BMI), definition and radiographic evaluation of liner malseating, clinical and functional outcomes among affected patients, as well as factors associated with malseating. Results were descriptively summarized as means and ranges in case of continuous variables, as well as percentages and absolute numbers for categorical variables.

## Results

**Fig. 1:**
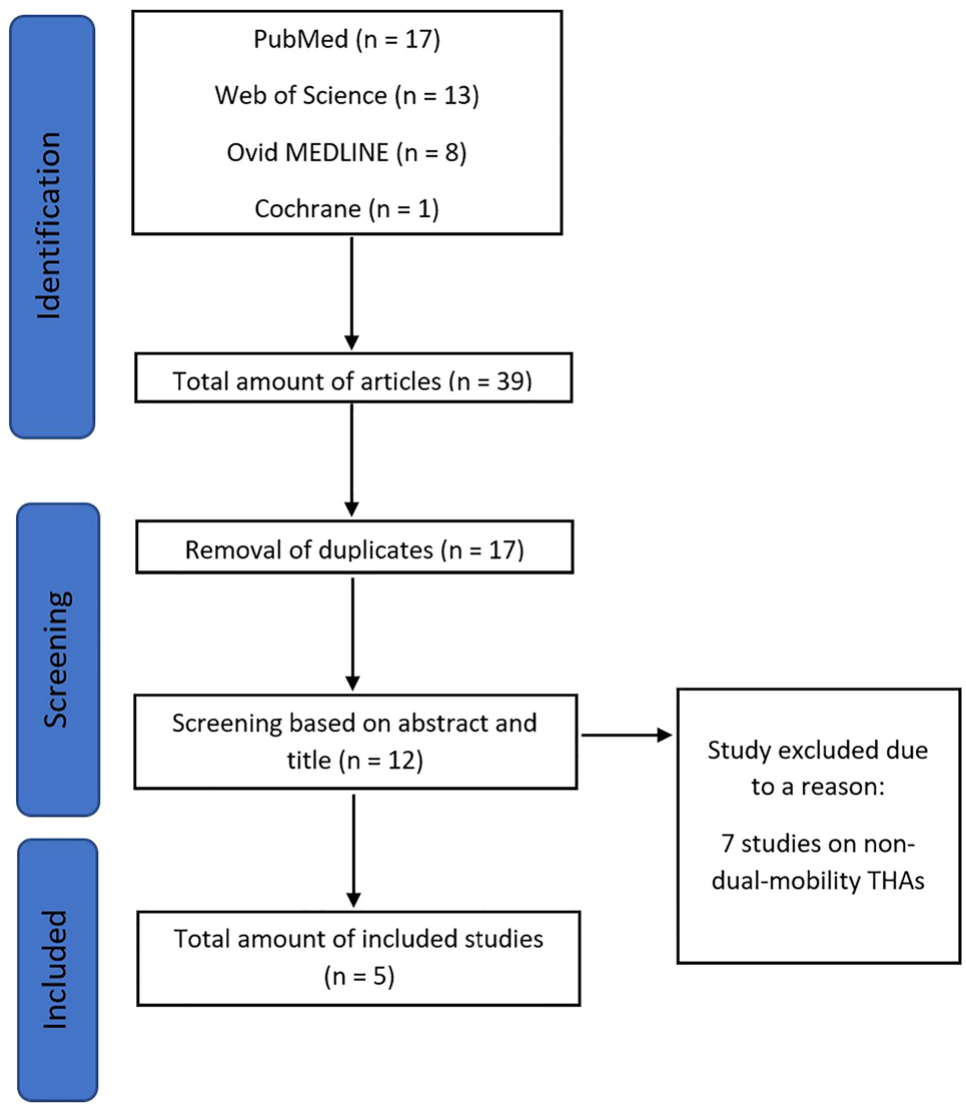
PRISMA based search of eligible studies

A total of 39 studies were identified based on PubMed (*n* = 17), Web of Science (*n* = 13), Ovid MEDLINE (*n* = 8), and Cochrane (*n* = 1) search (Fig. 1). After removal of duplicates, 17 articles were screened for study inclusion, and 12 records considered to be possibly eligible based on their title. After exclusion of 7 studies on non-dual-mobility THAs, 5 retrospective cohort studies, published between 2019 and 2021, were included in the final analysis (Table 1) [6, 12–15]. Except one multicenter study, all were single institution cohorts, and all conducted at major university-based US institutions between 2010 and 2020. Mean MINORS score was 9.8 (range, 8 to 11), and no study fulfilled level of evidence III or above.

**Table 1:** Liner malseating in dual-mobility THAs

	Guntin et al. [12]	Siljander et al., [13]	Salem et al. [14]	Chalmers et al. [15]	Romero et al. [6]
Journal	Bone and Joint Open	The Bone and Joint Journal	Surgical Technology International	Arthroplasty Today	The Bone and Joint Journal
Localization	Rush, Chicago, USA	Mayo Clinic, Rochester, USA	Lenox Hill Hospital, Mount Sinai West Hospital, and Hospital for Special Surgery, all New York, USA Sinai Hospital, Baltimore, USA	Hospital for Special Surgery, New York, USA	Hospital for Special Surgery, New York, USA
Study designs	Retrospective cohort, single institute	Retrospective cohort, single institute	Retrospective cohort, multicenter	Retrospective cohort, single institute	Retrospective cohort, single institute; experimental arm
Level of evidence	IV	IV	IV	IV	IV (clinical arm)
MINORS	8	11	10	11	9
Years of treatment	April 2011 to July 2020	January 2012 to December 2019	January 2010 to December 2018	2012 to 2018	January 2016 to December 2018
THAs (n)	239	256	1322	305	551
THA characteristics	•118 Stryker, USA (98 Trident I, 3 Trident II, 2 Trident II Tritanium, 1 primary Tritanium, 14 revision Tritanium components); 121 Zimmer Biomet, USA (121 Biomet G7) •Mean cup size 52.5 mm (range, 46 to 66) •54 times 22 mm femoral head, 185 times 28 mm	•144 Stryker, USA (124 Trident Tritanium, 15 Trident OST, 5 Trident); 112 Zimmer-Biomet G7 (55 G7 three-hole shells, 57 G7 Osseo TI multihole shells)	•941 primary THAs, 381 revision THAs •All MDM X3, Stryker, USA	•305 Stryker, USA (147 Trident I PSL (peripheral self-locking), 158 Trident II shells) •Median cup size 52 mm (range, 46 to 62 mm) •Cobalt-chrome liner (mean 42 mm (range, 36 to 52 mm)), polyethylene bearing •Median head size 42 mm (range, 36 to 52); 300 times 28 mm femoral heads (197 ceramic and 103 CoCr), 5 times 22.2 mm heads	•551 Stryker, USA (249 Trident I, 104 Trident PSL, 198 Trident II) •Mean cup size 52.5 mm (range, 44 to 60) •Cobalt-chrome liner
Patients F / M	219 (130/89)	233 (166/67)	1322	305 (149/156)	251 (12/239)
Age	65.8 years (range, 32 to 94)	66 years (range, 18 to 93)	Not evaluated	68 years (range, 31 to 92)	67.9 years (range, 28 to 95)
BMI	30.0 kg/m2 (18 to 57.4)	30.0 kg/m2 (17 to 57)	NA	31 kg/m2 (17 to 59)	28.3 kg/m2 (13.2 to 54.3)
Radiographic Follow-up	Not précised	10 (range, 0 to 66) and 6 months (range, 0 to 29) for Styker and G7 liners	Not précised	Minimum of 6 weeks, maximum 3 months	Minimum of 6 weeks
Liner malseating definition	•Stryker liner: gap between liner back and the rim of the acetabular shell or angulation between liner and shell •Biomet G7 liner: any distinct gaps on the flush implant surface	•Stryker liner: asymmetry or angulation between shell and liner •Biomet G7 liner: irregularity or disruption at the shell liner interface	Not given	Any measurable asymmetry	Gap between liner and shell or divergence between a line drawn along the face of the liner and the face of the shell
Linear Malseating	•8-times Stryker Trident I, 4-times G7 Zimmer Biomet •9-times females, 3-times males •9-times < 50.01 mm liner, 3-times ≥ 50.01	2-times Stryker, 1-times G7	2-times primary THA group	•3-times Trident I PSL, 1-time Trident II shell •2-times 50 mm cups, 2-times 52 mm •4-times 28-mm ceramic heads	•9-times Trident I, 5-times Trident PSL, 8-times Trident II •26 females, 6 males
	5% /12	1.2%/3	0.15%/2	1.3%/4	5.8%/32
Clinical Follow-up	14 months (range, 1.4 to 99.2)	3.5 years (range, 2.0 to 9.2)	Minimum 2 years	2 years (range, 1 to 5 years)	Maximum of 3 years in malseated group
Revision for liner malseating	No clinical consequences	No clinical consequence	No clinical consequence	No clinical consequence	No clinical consequence, one patient developed pain at two years
Metal ion	Normal serum metal ion levels preoperative, at 1 year and 2 years for one patient with a malseated Biomet G7 liner	Not collected	Normal serum metal ion levels at 5.3 and 7.1 years for the 2 patients with malseated liners	Not collected	Normal serum metal ion level for the one patient with malseated liner and pain
Functionality	No differences in Harris Hip Score (HHS), Hip disability and Osteoarthritis Outcome Score, and Veterans RAND 12-tem in malseated compared to non-malseated patients	HHS improved significantly from 46 (range, 24 to 69) preoperatively to 81 for both the Stryker and G7 groups	Not evaluated	Not evaluated	Not evaluated
Factors associated with malseating	Component size of 50 mm or less associated with malseating (univariate analysis, *p* = 0.031)	No significant difference between Stryker and G7	Not evaluated	Not evaluated	Females (*p* = 0.005), smaller cup sizes (*p*= 0.007), and lower BMI (*p* = 0.005) associated with malseating (not significant in multivariable analysis)

There were a total of 2330 patients (range, 219 to 1322) treated with 2673 dual-mobility THAs (2440 Stryker shells, 233 Zimmer Biomet shells). Mean age was 66.9 years (range, 65.8 to 68), mean BMI was 29.8 kg/m2 (range, 28.3 to 31 kg/m2), and 35% of patients were female (based on studies with available sex differentiation only).

**Fig. 2:**
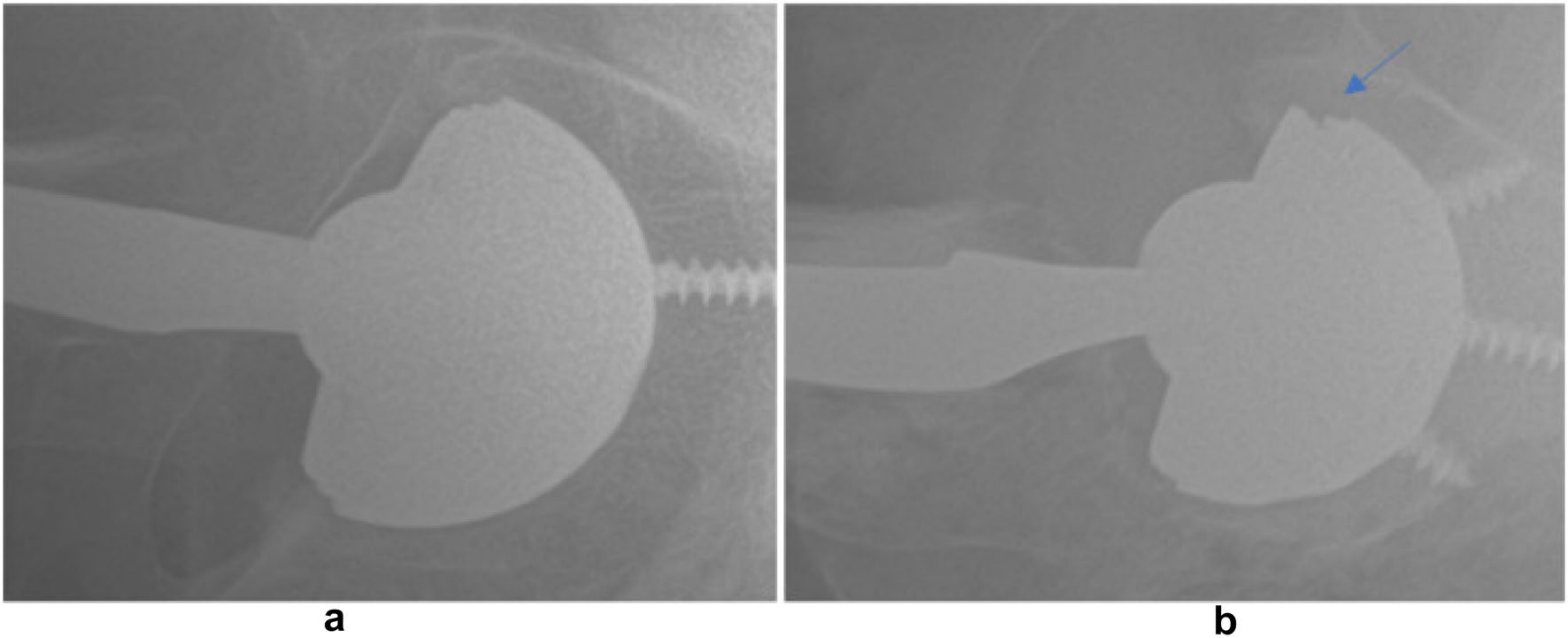
A well seated Stryker Trident liner (left) compared to a malseated liner with asymmetry and a gap (right) (from Guntin et al. [12] redistributed in accordance with CC BY-NC-ND 4.0. Open-access article redistributed in accordance with the terms of the Creative Commons Attribution Non-Commercial No Derivatives (CC BY-NC-ND 4.0) licence, which permits the copying and redistribution of the work only, and provided the original author and source are credited. See https://creativecommons.org/licenses/by-nc-nd/4.0/. Attribution—Appropriate credit was given, a link to the license is provided, no changes were made. NonCommercial—The material is not used for commercial purposes. NoDerivatives—The material was not remixed, transformed, or build upon. No additional restrictions—There are no legal terms or technological measures that legally restrict others from doing anything the license permits)

**Fig. 3:**
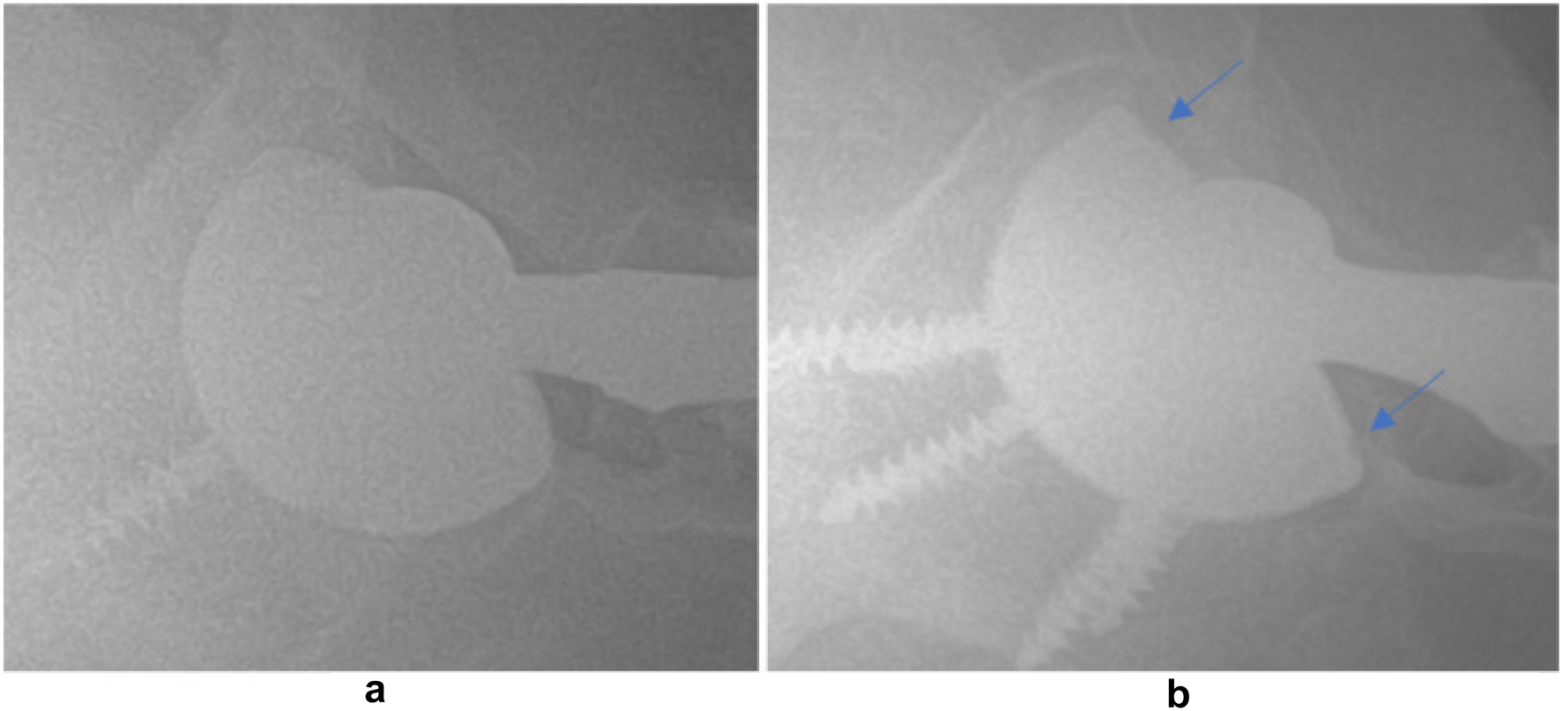
A well seated Zimmer Biomet G7 liner (left) compared to a malseated liner with gaps (right) (from Guntin et al. [12] redistributed in accordance with CC BY-NC-ND 4.0. Open-access article redistributed in accordance with the terms of the Creative Commons Attribution Non-Commercial No Derivatives (CC BY-NC-ND 4.0) licence, which permits the copying and redistribution of the work only, and provided the original author and source are credited. See https://creativecommons.org/licenses/by-nc-nd/4.0/. Attribution—Appropriate credit was given, a link to the license is provided, no changes were made. NonCommercial—The material is not used for commercial purposes. NoDerivatives—The material was not remixed, transformed, or build upon. No additional restrictions—There are no legal terms or technological measures that legally restrict others from doing anything the license permits)

In all but one study [14], linear malseating was evaluated by at least 2 independent reviewers, and at least a third reviewer used for confirmation and/or consensus. Patients without both an AP and cross table lateral radiograph were excluded in all studies. Radiographic follow-up was précised by 3 studies only, with two using a minimum of 6 weeks [6, 15], one reporting of a mean follow-up of 6 and 10 months for Stryker and Zimmer Biomet implants, respectively [13]. Liner malseating was defined in 4 studies, with all proposing a similar definition of gap, asymmetry, angulation, or disruption between liner and acetabular component [6, 12, 13, 15]. In total, 53 malseatings were identified among the 2673 THAs (1.98%), with the rates among studies ranging from 0.15% to 5.8% [6, 14]. Referred to implant type used, 48 malseatings occurred with Stryker (1.96%; Fig. 2) and 5 with Biomet Zimmer shells (2.14%; Fig. 3). Differentiation based on sex was performed in 2 studies, with 35 malseatings occurring in females, 9 in men [6, 12].

Clinical follow-up was described inconsistently among studies with 3 reporting of a mean follow-up (overall mean 2.2 years) [12, 13, 15], 4 including a minimum follow-up (overall mean 1.27 years) [12–15], and another 4 reporting of a maximum follow-up (overall mean 6.36 years) [6, 12, 13, 15]. Except one patient reporting of pain at 2 years [6], no clinical consequence was noted in the group of malseated liners, including no revision or negatively impacted functionality. Among patients with malseated liners, 4 patients had a metal ion measurement, all of them within a normal range [6, 12, 14]. Functionality was evaluated in 2 studies, with one demonstrating no differences between malseated and non-malseated liners [12].

Three studies performed a statistical analysis to identify factors associated with liner malseating. Guntin et al. [12] identified a component size of 50 mm or less to be associated with malseating. Likewise, Romero et al. [6] reported of a smaller cup size, as well as females, and a lower BMI as statistically significant risk factors, although no significance was noted in the course of a multivariable logistic regression analysis. Finally, Siljander et al. [13] could not show a significant difference between Stryker and Zimmer Biomet liners.

## Discussion

This is the first systematic review on liner malseating in patients undergoing dual-mobility THAs. We found a low rate of malseated liners (1.98%) in 2330 patients based on 5 different studies. Importantly, no revision or other negative clinical impact was noted in any of the aforementioned patients at short-term.

Liner malseating is not a new phenomenon and has previously been described with ceramic-on-ceramic liners. Of note, these prior reports demonstrated a substantially higher rate of malseating, ranging from 7.2% up to 25% at short-term [7, 16–19]. An explanation on this discrepancy is difficult, as the malseating mechanism itself is not understood in its entirety. Inferior interposition of soft tissue [6], deformation of acetabular components with under-reaming [20], and prominent screw heads are among the most common mechanisms discussed [15]. As such, the aforementioned discrepancy might be attributable to surgical factors, as suggested by Salem et al. [14]. Moreover, the authors believe low interobserver and intraobserver reliability [12], as well as studies limited to high-volume university centers only, to offer a potential explanation on differences in malseating rates between dual mobility and ceramic-on-ceramic THAs.

Malseating goes beyond a pure radiographic finding. Prior investigations on malseated ceramic-on-ceramic liners identified significant complications, including liner dissociation, metal fretting, implant interface motion, complete liner dislocation, liner fracture and penetration of the femoral head through the acetabular shell [8, 21–24]. In our analysis none of these complications were noted, although we acknowledge inconsistent and short-term follow-ups. Moreover, Romero et al. [6] identified earlier fretting onset compared with well-seated liners in the experimental arm of their study, suggesting possible long-term effects not analyzed in current investigations.

Factors associated with malseating are essential, as they might allow for risk factor modification before undergoing THA. In the course of our investigations, one study reported of smaller sized cups as a risk factor for malseating [12]. This is in contrast to findings on malseating in ceramic-on-ceramic THAs that identified higher age, reduced preoperative flexion, and THA for osteoarthritis, but not implant size, as significant risk factors [7]. No further factors, including sex, preoperative BMI, or implant design, were noted in our review, although analysis was limited by low event rates of malseated liners throughout.

This article had a number of limitations. Foremost included studies were retrospective cohort studies with none reaching level of evidence III or above. This was also reflected in a moderate quality assessed by two independent reviewers using the MINORS score. In addition, studies reported outcomes in one country only, including 3 studies from the same clinic (Hospital for Special Surgery) [6, 14, 15], limiting generalizability. Moreover, both radiographic and clinical follow-up were defined inconsistently throughout studies, and affected patients not followed up in detail. In specific, metal ions were only available in 4 cases, and functionality not compared to unaffected hips in all studies.

In conclusion, this systematic review found liner malseating to be a rare finding in dual-mobility THAs. While no direct clinical consequences were noted in any patient, a short-term clinical and inconsistent radiographic follow-up must be acknowledged. Detailed follow-up of affected liners at mid-term, including multiple radiographic controls, metal ion measurements, and functional assessments are necessary before drawing final conclusions.
